# The Relationship between Diabetes Mellitus and Gastric Cancer and the Potential Benefits of Metformin: An Extensive Review of the Literature

**DOI:** 10.3390/biom11071022

**Published:** 2021-07-13

**Authors:** Chin-Hsiao Tseng

**Affiliations:** 1 Department of Internal Medicine, National Taiwan University College of Medicine, Taipei 10051, Taiwan; ccktsh@ms6.hinet.net; Tel.: +886-2-2388-3578; 2 Division of Endocrinology and Metabolism, Department of Internal Medicine, National Taiwan University Hospital, Taipei 10051, Taiwan; 3 Division of Environmental Health and Occupational Medicine, National Health Research Institutes, Zhunan 350, Taiwan; 4 Department of Internal Medicine, National Taiwan University Hospital, No. 7 Chung-Shan South Road, Taipei 100, Taiwan

**Keywords:** diabetes mellitus, gastric cancer, *Helicobacter pylori*, insulin resistance, metformin, microbiota

## Abstract

The objective of this review is to summarize the findings of published research that investigated the relationship between diabetes mellitus and gastric cancer (GCa) and the potential benefits of metformin on GCa. Related literature has been extensively reviewed, and findings from studies investigating the relationship between diabetes mellitus and GCa suggest that hyperglycemia, hyperinsulinemia and insulin resistance are closely related to the development of GCa. Although not supported by all, most observational studies suggest an increased risk of GCa in patients with type 2 diabetes mellitus, especially in women and in Asian populations. Incidence of second primary malignancy diagnosed after GCa is significantly higher in diabetes patients. Diabetes patients with GCa may have more complications after gastrectomy or chemotherapy and they may have a poorer prognosis than patients with GCa but without diabetes mellitus. However, glycemic control may improve in the diabetes patients with GCa after receiving gastrectomy, especially after procedures that bypass the duodenum and proximal jejunum, such as Roux-en-Y gastric bypass or Billroth II reconstruction. The potential links between diabetes mellitus and GCa may involve the interactions with shared risk factors (e.g., obesity, hyperglycemia, hyperinsulinemia, insulin resistance, high salt intake, smoking, etc.), *Helicobacter pylori* (HP) infection, medications (e.g., insulin, metformin, statins, aspirin, proton pump inhibitors, antibiotics, etc.) and comorbidities (e.g., hypertension, dyslipidemia, vascular complications, heart failure, renal failure, etc.). With regards to the potential benefits of metformin on GCa, results of most observational studies suggest a reduced risk of GCa associated with metformin use in patients with T2DM, which can be supported by evidence derived from many in vitro and animal studies. Metformin use may also reduce the risk of HP infection, an important risk factor of GCa. In patients with GCa, metformin users may have improved survival and reduced recurrence. More studies are required to clarify the pathological subtypes/anatomical sites of GCa associated with type 2 diabetes mellitus or prevented by metformin, to confirm whether GCa risk can also be increased in patients with type 1 diabetes mellitus and to explore the possible role of gastric microbiota in the development of GCa.

## 1. Introduction

Throughout the world, although the incidence and mortality of gastric cancer (GCa) have been declining during the past decades, GCa remains the fifth most commonly diagnosed cancer and the fourth leading cause of cancer-related death [1,2,3,4]. Adenocarcinoma accounts for 95% of all cases of GCa [1]. It is estimated that more than 1 million new cases of GCa are diagnosed each year in the world, and the global age-standardized incidence and mortality rates for GCa are 11.1 and 8.2 per 100,000 people, respectively [2]. GCa is most commonly diagnosed between 55 and 80 years of age, and men have a 2- to 3-fold higher risk than women [2]. Several other risk factors of GCa have been identified, including genetic factors, *Helicobacter pylori* (HP) infection, gastric ulcer, gastroesophageal reflux disease, smoking, alcohol, chemical exposure, diet, obesity, pernicious anemia, gastric surgery, radiation, Epstein-Barr virus infection, socioeconomic status, blood group and race/ethnicity [1]. The prognosis is related to cancer staging and the average 5-year survival is 31% in the USA, 26% in Europe and 19% in the UK [1]. Screening programs of GCa can identify early cases, and HP infection screening with early eradication therapy may significantly affect the overall incidence and survival of GCa [1,2].

In Taiwan, according to the 2017 annual report of the Health Promotion Administration, Ministry of Health and Welfare, GCa is the 9th most common incident cancer (men: 7th and women 10th) [5]. The incidence of all GCa is also decreasing in Taiwan, from 15.97 per 100,000 population in 1996 to 11.57 per 100,000 population in 2013. Correspondingly, the incidence of gastric adenocarcinoma is decreasing, from 13.56 per 100,000 population in 1996 to 9.82 per 100,000 population in 2013 [6]. Eradication of HP infection is routinely performed for patients who are infected with HP in Taiwan, which significantly reduced the prevalence of HP infection from 54.4% in 1993 to 25.4% in 2014 and may have contributed to the decline of GCa in Taiwan [6]. Women generally have better survival than men, and the respective overall 3-year survival rates are 42.9% and 36.9% [6].

In 2004, we first demonstrated a significantly increased risk of cancer mortality by 28% in the diabetes patients in Taiwan while comparing the mortality of a large cohort of 256,036 diabetes patients to that of the Taiwan’s general population during the first 6 years of follow-up of the cohort [7]. Our subsequent epidemiological studies strongly supported an excess incidence of and mortality from various types of cancer including GCa in the diabetes patients [8,9].

Before 2010, only a few studies from Japan [10,11,12,13] and Korea [14,15] investigated GCa risk in association with diabetes mellitus [10,11,12,14] or fasting glucose [13,14,15], and the findings were contradictory and not conclusive. The large prospective follow-up study conducted in Korea published in 2005 suggested a significantly higher risk of GCa in the diabetes patients in terms of cancer incidence (hazard ratio (HR): 1.11, 95% confidence interval (CI): 1.04–1.20) and mortality (HR: 1.16, 95% CI: 1.04–1.28) in men but not in women [14].

In 2011, we first demonstrated in Taiwan an increased risk of mortality from GCa in the diabetes patients by following a large cohort of diabetes patients (113,347 men and 131,573 women) aged ≥ 25 years and recruited in 1995–1998 until the end of 2006 [8]. Mortality rate ratios comparing the diabetes patients to the average mortality rates of the general population consistently showed an excess mortality from GCa in the diabetes patients, which could be similarly shown in men and women but was most remarkable in the youngest age group of 25–64 years (mortality rate ratio (95% CI): 4.49 (3.93–5.12) for men and 3.65 (3.11–4.28) for women) [8]. After excluding patients who died of GCa within 5 years of diabetes diagnosis (to avoid the possibility of reverse causality), diabetes duration was a significant predictor for GCa mortality [8], suggesting a cause–effect relationship between diabetes mellitus and GCa.

Our subsequent study using the reimbursement database of Taiwan’s National Health Insurance (a nationwide healthcare system covering >99% of the population) showed a significant 14% higher risk of GCa in the diabetes patients while compared to the non-diabetes people [9]. At that time, because the mechanisms explaining such a close link between diabetes mellitus and GCa had not been researched, we proposed some putative mechanisms in a review article published in 2014 [16].

More than seven years have elapsed since we proposed the putative mechanisms linking diabetes mellitus and GCa, and many papers exploring the relationship among diabetes mellitus, metformin and GCa have emerged in the literature. To update the knowledge, the present article reviews, discusses and summarizes the findings of papers investigating the potential associations among diabetes mellitus, metformin and GCa under the following two sections: (1) The relationship between diabetes mellitus and GCa, and (2) potential benefits of metformin on GCa.

## 2. The Relationship between Diabetes Mellitus and Gastric Cancer

### 2.1. Hyperglycemia and Insulin Resistance Promote Gastric Cancer

GCa is characterized by altered glucose metabolism with upregulated aerobic glycolysis (Warburg effect) and has a high demand of glucose for cell growth and proliferation [17]. Studies conducted in cell cultures, in animals and in humans published in recent years support the concept that hyperglycemia and insulin resistance may promote the development of GCa.

Hyperglycemia can promote the development, proliferation, invasion and migration of cancer cells involving breast, liver, bladder, pancreas, colorectum and endometrium [18]. It can also promote human GCa cell proliferation and progression [19] and induce chemoresistance to 5-fluorouracil in GCa cells in an in vitro and in vivo study [20]. Zhou et al. showed that, in 978 patients with GCa, hyperglycemia with preoperative fasting plasma glucose ≥ 6.1 mM was associated with tumor size, location and late tumor stage [19]. The expression of aquaporin 3 (AQP3, a biomarker of GCa) was also increased in resected tumors in patients with higher fasting plasma glucose [19]. Additional in vitro studies conducted in human GCa cells of MGC803 and SGC7901 showed that the expression of AQP3 was upregulated by high glucose concentrations in a dose- and time-dependent manner [19]. The other in vitro and in vivo study by Zhao et al. suggested that hyperglycemia at 450 and 900 mg/dL would stimulate cancer cell proliferation and inhibit cancer cell response to the chemotherapeutic drug of 5-fluorouracil, compared to a glucose concentration of 100 mg/dL [20].

Human studies conducted in Japan suggested that fasting or 2 h post-load glucose was associated with the risk of GCa death [21] and that an A1C level ≥ 6.5% was associated with a significantly higher positive rate of stool occult blood [22]. Another Japanese study suggested that insulin, C-peptide and homeostasis model assessment of insulin resistance (HOMA-IR) but not glucose level were associated with an increased risk of GCa [23].

An early prospective follow-up study conducted in Korea did not show any significant association between GCa death or incidence with fasting serum glucose in either sex, except for GCa death in men with fasting serum glucose level 110–125 mg/dL compared to <90 mg/dL [14]. However, more recent Korean studies supported the findings of the Japanese studies by showing that fasting glucose, fasting insulin and insulin resistance indicated by HOMA-IR were associated with the risk of early GCa [24], and that high variability of fasting plasma glucose was independently associated with GCa in the diabetes-free population with either normal fasting glucose or impaired fasting glucose [25].

Therefore, evidence from recent studies suggest that hyperglycemia, hyperinsulinemia and insulin resistance may induce the development and proliferation of GCa.

Table 1 outlines the potential mechanisms explaining a higher risk of GCa in diabetes patients.

### 2.2. Diabetes Mellitus and Risk of Gastric Cancer

Type 2 diabetes mellitus (T2DM) is characterized by hyperglycemia and hyperinsulinemia resulting from insulin resistance. Therefore, theoretically, patients with T2DM may have an increased risk of GCa. Over the past 10 years, many papers have been published investigating the association between diabetes mellitus and GCa risk in humans. Most studies supported a positive link, though others might conclude a neutral association or even a reduced risk of GCa in the diabetes patients.

#### 2.2.1. Type 2 Diabetes Mellitus Increases Gastric Cancer Risk

An increased risk of GCa in the diabetes patients was supported by our previous studies [8,9] and by some later studies conducted by other investigators in Taiwan [26,27]. The excess mortality or increased risk of GCa has also been observed in patients with diabetes mellitus in many other different countries and ethnicities, including China [28,29,30,31,32], Hong Kong [33,34], Japan [23,35,36], Korea [37,38,39], the USA [40,41,42,43,44], Israel [45], Poland [46] and Finland [47].

Most of the above epidemiological studies included cases of GCa probably not well-documented by laboratory examinations or pathological reports. A recent retrospective cohort study conducted in Korea that followed 195,312 adult men and women with more accurate diagnosis by endoscopic examination confirmed such an increased risk of GCa in the diabetes patients, with an estimated adjusted HR of 1.76 (95% CI: 1.04–2.97) [48].

#### 2.2.2. Null Association in Some Studies

To a lesser extent, diabetes mellitus was not associated with GCa risk in some studies conducted in China [49], Korea [50], Israel [51], the USA [52], Sweden [53], Italy [54], France [55] and the UK [56].

A pooled analysis of over 771,000 individuals in the Asia Cohort Consortium with 19 prospective population-based cohorts showed a null association between diabetes mellitus and mortality from GCa (HR: 1.08, 95% CI: 0.95–1.23) [57]. However, when men and women were analyzed separately, an excess risk of GCa mortality could be seen in women (HR: 1.25, 95% CI: 1.03–1.53) but not in men (HR: 1.02, 95% CI: 0.90–1.17) [57]. These findings were somewhat different from our cohort study that showed an excess mortality from GCa in the diabetes patients disregarding sexes [8].

#### 2.2.3. Inverse Association

It is interesting that a few studies even showed a lower risk of GCa associated with diabetes mellitus or glucose load, including studies conducted in China [58], in Israeli Arabs [59] and in Italy [60].

#### 2.2.4. Meta-Analyses on Diabetes Mellitus and Gastric Cancer Risk

Several meta-analyses can be seen in the literature that investigated the risk of GCa associated with diabetes mellitus [57,61,62,63,64,65,66,67], prediabetes [68] and metabolic syndrome [69,70]. The main findings from meta-analyses on diabetes mellitus and GCa risk are summarized in Table 2 and discussed below.

The meta-analysis by Ge et al., that included 21 studies (4 case-control and 17 cohort studies) with cases of GCa incidence and mortality, suggested a null association between diabetes mellitus and GCa [61]. However, subgroup analyses in different sexes suggested an 18% higher risk in women (relative risk (RR): 1.18, 95% CI: 1.01–1.39) but not in men (RR: 1.04, 95% CI: 0.94–1.15) [61].

The meta-analysis by Tian et al. suggested a significantly higher risk of GCa in the diabetes patients in terms of cancer incidence (summary RR from 7 case-control studies and 14 cohort studies: 1.11, 95% CI: 1.00–1.24; summary RR in Asians derived from 6 studies: 1.19, 95% CI: 1.07–1.32) or mortality (summary RR from 7 studies: 1.29, 95% CI: 1.04–1.59) [62].

The meta-analysis by Shimoyama, including 12 studies, estimated an RR for GCa incidence of 1.41 (95% CI: 1.10–1.81) associated with diabetes mellitus [63]. Subgroup analyses suggested a greater magnitude of risk association in females than in males and the significant link was only observed in the Asian populations, but not in western populations [63].

The meta-analysis by Yoon et al., that included 17 studies (11 cohort studies and 6 case-control studies), suggested that pre-existing diabetes mellitus was associated with a significant 19% higher risk of GCa (RR: 1.19, 95% CI: 1.08–1.31), and this effect was not related to geographical regions [64]. Similarly, a significantly higher risk was observed in women but not in men [64].

The meta-analysis by Miao et al. included 22 cohort studies reporting incident or fatal cases of GCa and suggested that diabetes mellitus was not associated with overall changes in GCa, but certain subgroups, e.g., women of western countries (RR: 1.31, 95% CI: 1.09–1.57) and men aged < 55 years (RR: 1.16, 95% CI: 1.05–1.29), might show a significant risk association with GCa mortality [65]. The pooled analysis in Asian countries by Chen et al. also supported an excess mortality of GCa in women but not in men [57].

Ohkuma et al. analyzed the sex differences in the association between diabetes mellitus and cancer by including 121 cohorts that studied incident or fatal cases of cancer [66]. Among them, 27 reported GCa, and the estimated women-to-men ratio of RR for GCa was 1.14 (95% CI: 1.07–1.22), suggesting that women with diabetes mellitus had a significantly higher risk of GCa in comparison to their male counterparts [66]. Such an excess of women-to-men ratio of RR for GCa in the diabetes patients was also similarly observed in another meta-analysis published in the same year by Fang et al. [67]. This meta-analysis included 21 cohort studies and concluded a very similar women-to-men ratio of RR for GCa (1.14, 95% CI: 1.06–1.22) [67].

Another meta-analysis also suggested a significantly higher risk of cancer associated with prediabetes, especially cancer involving liver, endometrium and stomach/colorectum [68]. Since patients with prediabetes may not have been treated with antidiabetic drugs and they are less likely to suffer from diabetes-related complications, such a positive association implies a potential link between hyperglycemia and hyperinsulinemia resulting from insulin resistance before the diagnosis of T2DM in the pathogenesis of GCa.

A meta-analysis that investigated the risk of GCa comparing patients with metabolic syndrome versus those without metabolic syndrome (defined either by the criteria of the revised National Cholesterol Education Program’s Adults Treatment Panel III or by criteria of the International Diabetes Federation) concluded a null association [69]. Similarly, a later meta-analysis also concluded a null association between metabolic syndrome and GCa [70]. However, subgroup analysis in this later meta-analysis did show that western women with metabolic syndrome would have a significantly higher risk of GCa (HR 1.24, 95% CI: 1.05–1.47) [70]. The findings of these studies suggested that the different components of metabolic syndrome may not have the same impact on the risk of GCa and that the risk might not be the same in different subgroups of sex or ethnicities. These factors should be separately investigated in future studies.

It seems likely that the association between diabetes mellitus and GCa is more prominent in women and in Asian populations. The reasons for such discrepancies with regards to sex and ethnicity require more in-depth research, but there are some possible explanations. Hyperinsulinemia resulting from insulin resistance may increase the bioavailability of insulin-like growth factor (IGF)-1 by lowering IGF-binding proteins, and both insulin and IGF-1 can stimulate the growth of GCa [61]. Increased IGF-1 and insulin may also reduce the expression of sex hormone-binding globulin, leading to increased bioavailability of estrogen [63]. Estrogen receptor β can be expressed in stomach cells and its binding to estrogen may stimulate GCa cell proliferation [64]. Besides, differences in age distribution, diabetes duration, glycemic control, hormone replacement, etc., may also play some roles in the discrepancy between different sexes [66,67]. The difference between Asian and Western countries may represent different GCa incidences in different geographical regions with different environmental exposure, differences in dietary patterns or lifestyles, different HP infection rates, different genetic backgrounds, detection bias or confounding bias, etc. [62,63,67].

### 2.3. Outcomes and Prognosis in Patients with Gastric Cancer and Diabetes Mellitus

#### 2.3.1. Second Primary Malignancy Diagnosed after Gastric Cancer in Diabetes Patients

According to a 15-year follow-up study conducted in Taiwan, a total of 2110 second primary malignancies developed in 47,729 patients with GCa during 137,798 person-years of follow-up. Significantly higher standardized incidence ratios were observed for cancers of the head and neck, esophagus, colon and rectum, bones and soft tissue, ovaries, bladder, kidney and non-Hodgkin’s lymphoma. Diabetes mellitus was identified as an independent risk factor for the development of a second primary malignancy after the diagnosis of GCa, with an estimated HR of 1.30 (95% CI: 1.15–1.48) [75]. Therefore, close surveillance of patients with GCa, especially those with diabetes mellitus, is important for early diagnosis and intervention of a possible second primary malignancy.

#### 2.3.2. Deterioration of Hyperglycemia and Infection at Cancer Diagnosis

Some clinical studies suggested that patients with diabetes mellitus might develop poorer glycemic control, requiring the use of insulin [76], and have an increased risk of tuberculosis infection [77] when they are diagnosed with GCa.

#### 2.3.3. Poorer Prognosis

For patients with GCa, those who also suffered from diabetes mellitus were shown to have a poorer prognosis than those without diabetes mellitus in studies conducted in Taiwan [78], China [79,80], Iran [81] and the USA [82].

In Taiwan, the risk of 90-day mortality after gastrectomy for GCa is significantly higher in patients with diabetes-related complications (including eye disease, peripheral circulatory disorder, ketoacidosis, coma and renal disease, respectively) when compared to patients without diabetes mellitus, even though diabetes mellitus (either type 1 diabetes mellitus (T1DM) or T2DM) per se was not significantly associated with the prognosis [78].

In China, Wei et al. concluded in a retrospective cohort study that pre-existing diabetes mellitus was associated with more postoperative complications and worse survival [79]. Sheng et al. in a later study showed that the 3- and 5-year survival rates in 84 patients with GCa and diabetes mellitus were significantly lower than those observed in 84 propensity score-matched GCa patients without diabetes mellitus [80]. The 3-year survival rates were 38.1% and 65.5% (*p* = 0.004) respectively, and the 5-year survival rates were 32.1% and 52.4% (*p* = 0.023), respectively [80].

In Iran, Mohammadzadeh et al. used a decision tree model to identify factors affecting the mortality of patients with GCa [81]. They identified 9 important predictive variables, and diabetes mellitus ranked as the first in the order of importance [81].

The US study by Karlin et al. that included cancer cases of GCa or esophageal cancer also supported a significantly lower survival in patients with diabetes mellitus compared to non-diabetes patients [82]. The 3-year overall survival rates were 46% and 52%, respectively.

Studies conducted in Italy suggested that up to 56% of non-diabetes patients might develop hyperglycemia after gastric surgery for cancer [83]. They later showed that transient stress-induced hyperglycemia in the first 72 h after gastrectomy for GCa in non-diabetes patients may adversely affect the survival [84].

Since diabetes mellitus and hyperglycemia (even in non-diabetes patients) are important influential factors for survival in patients with GCa, it is important to appropriately control hyperglycemia in patients with or without diabetes mellitus after gastrectomy. Furthermore, it is worthy to point out that the risk association estimated from incident cases may differ from that estimated from mortality cases. Therefore, an excess mortality from GCa in the diabetes patients may not necessarily imply an excess incidence of GCa in the diabetes patients. Future studies should separately consider the inclusion of incident cases and mortality cases. Despite these potential differences, current evidence supports an association between diabetes mellitus and GCa in terms of either incidence or mortality.

#### 2.3.4. Remission of Diabetes Mellitus after Gastrectomy

When patients with diabetes mellitus and GCa receive gastrectomy, studies suggested that they may have improved glycemic control. This finding has been consistently observed in many clinical studies conducted in different ethnicities and different regions of the world [79,85,86,87,88,89,90,91,92,93,94,95]. Studies including a meta-analysis suggested that Billroth II reconstruction (gastrojejunostomy) is more effective than Billroth I (gastroduodenostomy) in the improvement of glycemic control [96,97,98,99]. Although not yet fully confirmed, there are some hypotheses that can explain the improvement of hyperglycemia after gastrectomy and the better effect of diabetes remission associated with Billroth II reconstruction [96]. The foregut theory suggests that some putative signals leading to insulin resistance can be removed by the excision of duodenum and the proximal jejunum [96]. The hindgut theory suggests that accelerated delivery of nutrients to the hindgut exaggerates the secretion of gut hormones such as glucagon-like peptide-1 and peptide YY from the L-cells of ileum, both having an anorexigenic effect [96]. Furthermore, ghrelin, another gut hormone produced mainly by the X/A cells of the stomach that has actions of stimulating appetite and food intake, is decreased after gastrectomy [99]. As a result, postprandial insulin secretion is increased by glucagon-like peptide-1, appetite and food intake are reduced by the changes of gut hormones and insulin resistance is reduced with the loss of body weight and the removal of part of the foregut. Gastrojejunostomy that bypasses both the duodenum and the proximal part of the jejunum facilitates a rapid delivery of nutrients to the distal intestine and leads to an exaggerated secretion of gut hormones from the L-cells. This could be one of the reasons explaining a better outcome in terms of glycemic control observed in surgical procedures that involve gastrojejunostomy.

A Korean study showed that diabetes mellitus was cured in 15.1% and improved in 30.4% of the patients with T2DM after gastrectomy for GCa [100]. Body mass index (BMI) reduction ratio was found to be the most influential factor for metabolic improvement [100]. Billroth I had the lowest BMI reduction ratio and Roux-en-Y gastric bypass had a greater BMI reduction ratio than Billroth II [100]. A study conducted in China suggested that the glycemic changes after Billroth II gastrojejunostomy might differ according to the baseline glycemic status of T2DM/impaired glucose tolerance and normal glucose tolerance in non-obese patients with GCa [101]. While patients with T2DM and impaired glucose tolerance might have a reduction of blood glucose, those with normal glucose tolerance would have deteriorated blood glucose in a 3-month time period [101]. Currently, there is no explanation for such contradictory responses, but the investigators postulated an involvement of the alteration of gut hormones. Recent studies also supported that the remission of diabetes mellitus and improvement of metabolic control might be related to the changes of gut microbiota after Billroth II or Roux-en-Y gastric bypass [102,103].

#### 2.3.5. More Complications after Gastrectomy or Chemotherapy in Diabetes Patients

More complications may develop in diabetes patients with GCa after surgical operation or after chemotherapy [104,105,106], especially in the elderly [107]. However, diabetes mellitus was not observed to be a risk factor for postoperative complications after gastrectomy in patients with GCa in another Japanese study [108].

Studies conducted in Korea suggested an increased risk of gallstone disease [109,110] and anemia [111] in patients with diabetes mellitus who receive gastrectomy for GCa.

Studies conducted in China suggested that diabetes patients may have prolonged stays in intensive care units [112] and an increased risk of intra-abdominal infection [113] and unplanned reoperation [114] after gastrectomy for GCa. A US study also showed that the 30-day readmission rate was higher in diabetes patients after gastrectomy [115], and the higher readmission rate in the diabetes patients was confirmed in a meta-analysis including 6 studies [116].

Studies conducted in Korea [117] and Japan [118] suggested that diabetes patients may have a significantly higher risk of osteoporosis and fracture after gastrectomy for GCa.

The risk of postoperative pulmonary infection is increased in patients with diabetes mellitus in studies conducted in Japan [119] and China [120].

A meta-analysis suggested that diabetes patients would have an increased risk of anastomotic leakage after gastrointestinal surgery [121].

The probability of getting other primary cancers is higher in diabetes patients than non-diabetes patients who received surgical treatment for their GCa [122].

Studies conducted in Japan showed that thrombocytopenia associated with oxaliplatin treatment for advanced GCa was more frequently observed in patients with diabetes mellitus [123] and that the risk of acute kidney injury is increased in diabetes patients who receive chemotherapy for their GCa [124].

The outcomes and prognosis in patients with GCa and diabetes mellitus are summarized in Table 3.

### 2.4. Potential Links between Diabetes Mellitus and Gastric Cancer

We proposed some putative mechanisms linking diabetes mellitus and GCa a few years ago [16]. These potential links may involve the interactions with shared risk factors (e.g., obesity, hyperglycemia, hyperinsulinemia, insulin resistance, high salt intake, smoking, etc.), HP infection, medications (e.g., insulin, metformin, aspirin, statins, proton pump inhibitors, antibiotics, etc.) and comorbidities (e.g., hypertension, dyslipidemia, vascular complications, heart failure, renal failure, etc.) [16]. Furthermore, screening programs may also affect the detection and mortality rates of GCa associated with diabetes mellitus. These potential links and the effects of screening programs are summarized in Table 1.

Evidence of the stimulating effects of hyperglycemia, hyperinsulinemia and insulin resistance on the growth and proliferation of GCa cells has emerged from recent cellular and epidemiological studies, as discussed earlier in this article. Some other factors affecting the links that have been more vigorously researched in recent years are discussed below, with regards to (1) HP infection, (2) insulin, (3) metformin, (4) aspirin, statin and proton pump inhibitors and (5) screening.

#### 2.4.1. Helicobacter Pylori Infection

HP infection is a well-known risk factor of GCa, and eradication of HP infection reduces the risk of GCa [6,125]. Patients with T2DM may suffer from a higher risk of HP infection, as shown in our previous observational study [126]. A meta-analysis also suggested that diabetes patients may have a higher risk of failing eradication of HP infection [127]. A cellular study supported a role of hyperglycemia in maintaining the growth and viability of HP [128], providing evidence for the close link between diabetes mellitus and HP infection. Therefore, HP infection will surely play some role in the association between diabetes mellitus and GCa. Complete eradication of HP infection can also be expected to reduce the risk of GCa in the diabetes patients.

#### 2.4.2. Insulin

The use of antidiabetic drugs and the control of hyperglycemia may modify the risk of GCa in the diabetes patients. The two most commonly studied antidiabetic drugs in relation to cancer risk are insulin and metformin, but some studies also investigated the effects of other drugs.

The development and proliferation of GCa may involve a wide variety of molecular targets on the cell membrane, such as hepatocyte growth factor receptor, platelet-derived growth factor receptor, vascular endothelial growth factor receptor, human epidermal growth factor receptor-1, human epidermal growth factor receptor-2 and IGF-1 receptor (IGF-1R) [129,130]. Insulin receptor (IR) [131] and IGF-1 and IGF-2 [132] are highly expressed in GCa cells. Insulin and IGF-1 are closely related proteins, and binding of insulin to IR isoform A (IR-A: differs from IR-B with deletion of exon 11 from IR-B and has a high affinity to insulin and IGF-2) or hybrid IR/IGF-1R can trigger tumorigenesis [133,134,135,136]. Insulin resistance and hyperinsulinemia may lead to an over-stimulation of the tumorigenic pathways, leading to the development of GCa [135]. IGF-1 has a high affinity to IGF-1R and hybrid receptors of IR (A or B)/IGF-1R, and IGF-2 has a high affinity to IR-A, IR-A/IGF-1R and IGF-1R [135]. Insulin, either from exogenous injection or from endogenous hyperinsulinemia as a result of insulin resistance, together with the overexpressed IGF-1 and IGF-2 (more remarkably expressed) from occult cancer cells or the local production of IGF-2 in response to hypoxia in the tissue [136], can theoretically promote the growth of GCa cells via their activation of mitogenic pathways. The IGF-2/IR-A signaling is considered a major booster for malignancy in patients with diabetes or prediabetes [136].

Whether exogenous insulin used for hyperglycemic control in the diabetes patients may cause an increased risk of cancer, including GCa, is an important clinical issue that has been researched for years. A study conducted in the Danish population suggested a potential link between insulin use and GCa [137]. This was supported by another Taiwanese study [138] and a meta-analysis [139]. Although continuous follow-up of our diabetes cohort [7] for up to 17 years supported that the use of insulin is independently predictive for all-cancer death [140], we were not able to show any association between insulin use and GCa death [8] or GCa risk [9]. In clinical practice, it is wise to use insulin for glycemic control at the lowest optimal dose so as to avoid hypoglycemia and excess insulin in the blood.

#### 2.4.3. Metformin

Metformin was shown to reduce cancer risk in patients with T2DM in a recent meta-analysis [141]. In Taiwan, approximately 10 years ago, we first demonstrated that patients with T2DM treated with metformin for 3 or more years would have a reduced risk of colon cancer (HR: 0.646, 95% CI: 0.490–0.852) [142]. Since then, the anti-cancer effect of metformin has been demonstrated for other types of cancer in Taiwan, including breast cancer [143], thyroid cancer [144], bladder cancer [145], prostate cancer [146], endometrial cancer [147], ovarian cancer [148], cervical cancer [149], kidney cancer [150], oral cancer [151], esophageal cancer [152], lung cancer [153], colorectal cancer [154], nasopharyngeal cancer [155], skin cancer [156], pancreatic cancer [157], hepatocellular cancer [158], biliary tract cancer [159], non-Hodgkin’s lymphoma [160] and GCa [161]. An overall risk reduction of approximately 50% in GCa was consistently observed in metformin users when compared to never users of metformin after a follow-up duration of approximately 5 years [161]. However, it is admitted that this benefit of metformin on GCa remains unsettled [162] and more details will be discussed later under the subtitle of “3.1. Metformin and risk of gastric cancer in diabetes patients”.

#### 2.4.4. Aspirin, Statins and Proton Pump Inhibitors

Aspirin and statins may reduce the risk of GCa, but proton pump inhibitors have been shown to increase the risk [163,164,165,166,167,168]. However, a recent study conducted in Korea suggested that aspirin use was associated with a reduced incidence of and mortality from GCa, but metformin and statins were only associated with a reduced mortality from GCa [169].

#### 2.4.5. Screening

Implementation of a screening program may lead to early detection of GCa, and therefore a detection bias resulting from screening may affect the estimation of risk association in epidemiological studies. On the other hand, intervention of patients with early cancer can affect the prognosis of these screen-detected patients. A Korean study suggested that fewer diabetes patients would receive recommended GCa screening than non-diabetes people (38.9 vs. 42.9%, *p* < 0.001) [170]. Therefore, the different screening rates between diabetes patients and non-diabetes people would surely affect the risk estimation with regards to incidence or mortality.

## 3. Potential Benefits of Metformin on Gastric Cancer

### 3.1. Metformin and Risk of Gastric Cancer in Diabetes Patients

Metformin has been recommended as the first-line therapy for patients with T2DM since 2012 by the American Diabetes Association and the European Association for the Study of Diabetes because of its multiple beneficial effects, beyond glucose lowering [171]. It is estimated that more than 150 million people in the world are prescribed metformin annually [172,173]. Its potential usefulness in lowering cardiovascular diseases was first recognized in the United Kingdom Prospective Diabetes Study in 1998 [174]. Since then, metformin has been shown to exert pleiotropic effects of anti-atherosclerosis, anti-cancer, anti-aging, anti-microbia, anti-inflammation and immune modulation [175,176,177,178]. Recently, by using the nationwide database of the National Health Insurance in Taiwan, we also showed that metformin use in patients with T2DM was associated with a lower risk of the following diagnoses: hypertension [179], hospitalization for heart failure [180], hospitalization for atrial fibrillation [181], chronic obstructive pulmonary disease [182], varicose veins [183], hemorrhoids [184], dementia [185,186], nodular goiter [187], uterine leiomyoma [188], osteoporosis/vertebral fracture [189] and inflammatory bowel disease [190].

Most studies investigating GCa risk associated with metformin use reported a beneficial effect. However, some reported a null association. These reports are discussed below with regards to their conclusions.

#### 3.1.1. Metformin Reduces Risk of Gastric Cancer

Metformin was shown to reduce GCa in observational studies in Taiwan [161], Korea [163,191], Italy [192] and Lithuania [193].

In a cohort study conducted in Hong Kong, metformin also reduced the incidence of GCa among HP-eradicated diabetes patients, independent of A1C level [194].

#### 3.1.2. Null Effect of Metformin

An early observational study in Taiwan did not show a lower risk of GCa associated with metformin use [195]. Some later studies also reported a null association, including studies conducted in the USA [196], Sweden [197] and the Netherlands [198].

#### 3.1.3. Meta-Analyses on Metformin and Gastric Cancer risk

Several meta-analyses have been conducted to investigate the effect of metformin on GCa risk, and most of them reported a beneficial effect. The main findings of these meta-analyses are summarized in Table 2 and discussed below.

An early meta-analysis by Franciosi et al., that included only 2 cohort studies, reported that metformin use was associated with a lower risk of GCa, with an estimated HR of 0.83 (95% CI: 0.76–0.91) [71].

A later meta-analysis by Zhou et al. including 7 cohort studies estimated a pooled HR of 0.763 (95% CI: 0.642–0.905) and found that studies conducted in Taiwan showed a more remarkable risk reduction (HR: 0.514, 95% CI: 0.384–0.688) [72].

Two recent meta-analyses showed risk reduction associated with metformin use, though the HRs were not statistically significant. The meta-analysis by Li et al. included 4 cohort studies and estimated a HR of 0.867 (95% CI: 0.726–1.035) [73], and the meta-analysis by Shuai et al. estimated a HR of 0.790 (95% CI: 0.624–1.001) [74]. Shuai et al. also showed that the reduced risk was especially significant in Asian populations but not in western populations [74].

### 3.2. Metformin and Survival of Patients with Gastric Cancer

Metformin was shown to improve survival and reduce recurrence of GCa in patients with T2DM in studies conducted in Belgium [199], Korea [200,201] and Taiwan [202]. However, this survival benefit of metformin for GCa was not similarly observed in studies conducted in Shanghai, China [203], and in Guangzhou, China [204]. In studies conducted in Lithuania, even though metformin use was associated with a reduced risk of GCa [193], its use did not affect the survival of patients with T2DM and GCa [205].

Though not statistically significant, a meta-analysis suggested an improvement of overall survival and cancer-specific survival by approximately 20% among metformin users in patients with GCa [74].

### 3.3. Potential Mechanisms of Metformin Against Gastric Cancer

The potential mechanisms explaining the general anti-cancer effects of metformin have been extensively reviewed [179,206,207]. Metformin may also inhibit cancer invasion and migration [208]. Interested readers may refer to some recent review articles that discuss the potential usefulness and mechanisms of metformin on GCa [209,210].

Table 4 summarizes the results of many cellular studies that researched the effects of metformin in GCa cells and suggested the involvement of many potential pathways. Metformin may suppress GCa via the inhibition of a signaling pathway involving hypoxia-inducible factor 1α/M2 isoform of pyruvate kinase [211], through a 5′ adenosine monophosphate-activated protein kinase (AMPK)-dependent inhibition of the Shh signaling pathway [212], through suppression of metastatic traits of epithelial–mesenchymal transition (EMT) [213], through stimulating calmodulin-like protein 3 secretion from gastric tumor-associated fibroblasts [214], via promoting beclin 1-dependent autophagy [215], by triggering the intrinsic apoptotic response via activating AMPK and suppressing mammalian target of rapamycin (mTOR)/protein kinase B (mTOR/Akt) signaling [216], by targeting GCa stem cells [217], by inhibiting angiogenesis and metastasis involving the protein tyrosine phosphatase receptor delta-C-X-C motif chemokine ligand 8 axis [218], by inhibiting invasion via downregulating long non-coding RNA H19 [219], through increasing acid-secreting parietal cells from stem cells via AMPK-dependent pathways [220], by inhibiting the transcription factor hepatocyte nuclear factor 4 alpha (HNF4α) via downregulation of *KLF5/GATA4/GATA6* oncogenes [221], through inducing apoptosis via inhibition of survivin mediated by AMPK/mTOR [222], via blocking cell cycle in G_0_–G_1_ in vitro and in vivo by decreasing G_1_ cyclins and reducing the phosphorylation of epidermal growth factor receptor and IGF-1R, probably via altering miRNAs expression [223], via modulating metastatic capacity by reducing the expression of vascular endothelial growth factor and blocking EMT [224], through decreasing the expression of EMT and stemness markers and reducing spheroid formation [225], via increasing the expression of the phosphorylated form of acetyl-CoA carboxylase [226], by downregulating specificity protein transcription factors [227], via the AMPKα-HNF4α-WNT5A signaling cascade [228], by suppressing HP-induced apoptosis [229], by downregulation of B-lymphoma Moloney murine leukemia virus insertion region-1, an oncogene, via AMPK [230], and by activating calcium binding protein 39-like, a tumor suppressor gene, via AMPK [231].

Metformin also inhibits in vivo tumor xenograft metastasis to peritoneum, probably via inhibition of nuclear factor-ĸB expression [232]. In db/db mice, metformin inhibits N-methyl-N-nitrosourea-induced GCa development [233].

Metformin may also have an interaction with chemotherapeutic drugs. An early cellular study using the human gastric MKN-45 cell line showed an antagonistic effect of metformin on cisplatin [234]. However, later studies suggested a synergistic effect of metformin and chemotherapeutics, including cisplatin [227] and cisplatin, adriamycin and paclitaxel [235]. Another recent study also showed that metformin may sensitize GCa cells to chemotherapeutic drugs, including 5-fluorouracil and docetaxel [236]. The synergistic effect of metformin and cisplatin or rapamycin was also demonstrated in an animal study using mice [237].

It is interesting that we have also demonstrated a significantly lower risk of HP infection associated with metformin use in patients with T2DM [238].

In summary, metformin may reduce the risk of GCa by inhibiting GCa cell growth, invasion, migration and metastasis via its various cellular actions. It may also reduce the risk of GCa by reducing related risk factors such as HP infection. Additionally, metformin reduces insulin level, improves insulin resistance and exerts anti-inflammatory effects [239]. All of these may contribute to its preventive and therapeutic effects on GCa.

## 4. Future Perspectives

Some unanswered questions regarding the relationship between diabetes mellitus/metformin and GCa are discussed as follows.

### 4.1. Association with Regards to Pathological Subtypes and Anatomical Sites Remains to Be Clarified

The association between diabetes mellitus/metformin protection and GCa pathological subtypes or anatomical sites has not been extensively explored. Adenocarcinoma represents 95% of GCa [1], and therefore most epidemiological studies might have included cases of adenocarcinoma. Whether the association between diabetes mellitus and other subtypes of GCa such as primary gastric lymphoma is not the same as for adenocarcinoma is not known. Cardiac and non-cardiac GCa may have different risk factors, and their incidence rates also vary in different geographical regions [2]. GCa related to HP infection is mainly non-cardiac in anatomical sites, and 90% of cases of non-cardiac origin can be attributed to HP infection [1]. Whether the protective effects of metformin on cardiac and non-cardiac GCa can be different remains to be clarified.

### 4.2. Is There a Link between Type 1 Diabetes Mellitus and Gastric Cancer?

As previously pointed out [16], most observational studies investigating the risk of GCa associated with diabetes mellitus did not discern between the two types of diabetes mellitus, i.e., T1DM and T2DM. However, it is believed that most of the studies included a vast majority of the patients with T2DM because T2DM greatly outnumbers T1DM in different regions of the world. A recent meta-analysis that included 9 studies with 256 cases of GCa estimated a significantly higher risk of GCa in patients with T1DM (RR: 1.41, 95% CI: 1.20–1.67) [240].

The mechanisms underlying the increased risk of GCa in patients with T1DM are worth some discussion. First, because insulin is a life-saving drug in patients with T1DM and the carcinogenic effect of insulin analogs may differ from that of human insulin [241,242,243], it remains to be explored whether the high incidence of GCa in these patients could be associated with the high frequency of the use of insulin analogs with mitogenic activity. Second, T1DM is an autoimmune disease characterized by immune-mediated destruction of insulin-secreting pancreatic β cells. GCa is not only significantly associated with T1DM but also a wide variety of other autoimmune diseases, including dermatomyositis, pernicious anemia, Addison’s disease, dermatitis herpetiformis, IgG4-related disease, primary biliary cirrhosis, systemic lupus erythematosus and Graves’ disease [240]. The mechanisms underlying the link between autoimmune diseases and GCa may be related to the inflammation resulting from immune-mediated destruction of the stomach or through gastritis driven by HP infection, which can be exacerbated in the presence of autoimmune diseases and the use of immunosuppressants [240]. Third, another possible link is the greater severity and impact of hyperglycemia that cannot be protected by the use of metformin, an oral antidiabetic drug that is approved only for patients with T2DM but not for patients with T1DM. Therefore, the underlying mechanisms for the increased risk of GCa in patients with T1DM requires more detailed research. The elucidation of the underlying mechanisms provides a possible point for GCa protection and intervention in these patients with T1DM.

### 4.3. Can Gastric Microbiota Play a Role?

Recently, some investigators proposed a role of gastric microbiota in the development of GCa [244]. However, except for HP infection, we do not currently have strong evidence to support such a role of other gastric microbiota in the development of GCa [245]. Metformin may affect intestinal microbiota, which contributes to its multiple beneficial effects [246,247], but whether metformin may affect the compositional change of gastric microbiota is an interesting topic that has rarely been researched. The roles of gastric microbiota and their interactions with intestinal microbiota in the development of GCa await further investigation.

## 5. Conclusions

Patients with abnormal glucose metabolism, especially T2DM, may have a high risk of GCa in terms of incidence or mortality. The association between diabetes mellitus and GCa is more remarkable in women and in Asian populations. The link between T2DM and GCa can be mediated by hyperglycemia, insulin resistance, shared risk factors, medications, comorbidities, high salt intake or higher HP infection rate. GCa patients with diabetes mellitus would have more complications and a poorer prognosis than GCa patients without diabetes mellitus. However, glycemic control may improve, and remission of diabetes mellitus can be seen in diabetes patients after gastrectomy, especially in patients who receive surgical procedures that bypass duodenum and the proximal jejunum. Metformin exerts anti-cancer effects in GCa cells, and observational studies do show a lower risk of GCa associated with metformin use in patients with T2DM and an improved survival with reduced recurrence in GCa patients who use metformin. Some future perspectives with regards to the pathological subtypes and anatomical sites of GCa associated with T2DM or prevented by metformin, the link between T1DM and GCa and the role of gastric microbiota in the development of GCa remain to be explored.

## Figures and Tables

**Table 1:** Potential mechanisms explaining a higher risk of gastric cancer in diabetes patients.

Potential Mechanisms	Explanations
**I.** **Direct effect**	
Hyperglycemia and insulin resistance promote GCa	In in vitro and in vivo studies, high glucose levels promote GCa cell growth and proliferation and induce chemoresistance to 5-fluorouracil. Insulin resistance and hyperinsulinemia may lead to an over-stimulation of the tumorigenic pathways of insulin, leading to the development of GCa. In human epidemiological studies, high glucose levels or insulin resistance biomarkers are associated with an increased incidence of or mortality from GCa. Some meta-analyses suggest a significantly higher risk of GCa in diabetes patients and in people with prediabetes.
**Ⅱ.** **Indirect links**	
Shared risk factors	GCa and type 2 diabetes mellitus share some important risk factors, such as obesity, hyperglycemia, hyperinsulinemia, insulin resistance, smoking etc.
2. Salt intake	High salt intake is a potential risk factor of GCa. Diabetes patients may have loss or impairment of taste, leading to a higher intake of salt. On the one hand, diabetes patients may have been advised to restrict salt intake because of heart or kidney diseases.
3. *Helicobacter pylori* infection	*Helicobacter pylori* infection is known to cause GCa. A cellular study supports the role of hyperglycemia in maintaining the growth and viability of *Helicobacter pylori.* Diabetes patients are prone to have a higher infection rate and a lower eradication rate of *Helicobacter pylori* infection.
4. Medications	Some drugs commonly used by diabetes patients may affect GCa risk. Metformin, aspirin and statins are associated with a lower risk of GCa. Insulin and proton pump inhibitors are associated with a higher risk of GCa. Antibiotics may affect the infection rate of *Helicobacter pylori.*
5. Comorbidities	Comorbidiites such as hypertension and dyslipidemia are also associated with insulin resistance. Patients with vascular complications such as heart failure and renal failure may have changed their lifestyle, daily activity, salt intake and dietary pattern. Patients with liver or kidney disease may have altered drug metabolism and they may be treated with medications that affect GCa risk. Detection bias because of a higher tendency to receive extra laboratory examination.
**Ⅲ.** **Screening effect**	
Screening program	A Korean study suggests that diabetes patients are less likely to receive GCa screening. Screening can lead to a higher incidence but early intervention may reduce the mortality rate.

GCa: gastric cancer.

**Table 2:** Main findings from meta-analyses on the relationship between diabetes mellitus and gastric cancer and on the effect of metformin on gastric cancer.

Authors	Year Published	Studies Included in Meta-Analysis	Outcome of Gastric Cancer	Estimated Relative Risk (95% Confidence Interval)	References
**Risk** **of gastric cancer associated with** **diabetes mellitus**					
Ge et al.	2011	4 case-control and 17 cohort studies	Incidence and mortality	All: 1.09 (0.98–1.22) Women: 1.18 (1.01–1.39) Men: 1.04 (0.94–1.15)	[61]
Tian et al.	2012	7 case-control and 14 cohort studies	Incidence	All: 1.11 (1.00–1.24) Case-control: 1.04 (0.84–1.29) Cohort: 1.14 (1.01–1.30)	[62]
		6 studies from Asia	Incidence	Asian: 1.19 (1.07–1.32)	
		7 studies	Mortality	1.29 (1.04–1.59)	
Shimoyama	2013	2 case-control and 10 cohort studies	Incidence	All: 1.41 (1.10–1.81)	[63]
		6 studies		Men: 1.24 (1.08–1.43) Women: 1.90 (1.27–2.85)	
		5 studies		Asian: 1.77 (1.38–2.26)	
		6 studies		White: 1.23 (0.90–1.68)	
Yoon et al.	2013	6 case-control and 11 cohort studies	Incidence	All: 1.19 (1.08–1.31) Men: 1.10 (0.97–1.24) Women: 1.24 (1.01–1.52) Asian: 1.19 (1.02–1.38) Western: 1.18 (1.03–1.36)	[64]
Miao et al.	2017	15 cohort studies	Incidence	1.10 (0.94–1.29)	[65]
		11 cohort studies	Incidence	Men: 1.00 (0.90–1.11)	
		10 cohort studies	Incidence	Women: 1.07 (0.93–1.22)	
		9 cohort studies	Mortality	All: 1.28 (0.93–1.76) Men < 55 years old: 1.16 (1.05–1.29) Western women: 1.31 (1.09–1.57)	
Chen et al.	2017	19 cohorts in Asian countries	Mortality	All: 1.08 (0.95–1.23) Men: 1.02 (0.90–1.17) Women: 1.25 (1.03–1.53)	[57]
Ohkuma et al.	2018	27 cohort studies	Incidence and mortality	Women-to-men ratio: 1.14 (1.07–1.22)	[66]
Fang et al.	2018	21 cohort studies	Incidence and mortality	Women-to-men ratio: 1.14 (1.06–1.22)	[67]
**Effect of metformin on gastric cancer**					
Franciosi et al.	2013	2 cohort studies	Incidence	0.83 (0.76–0.91)	[71]
Zhou et al.	2017	7 cohort studies	Incidence	0.763 (0.642–0.905)	[72]
Li et al.	2018	4 cohort studies	Incidence	0.867 (0.726–1.035)	[73]
Shuai et al.	2020	11 cohort studies	Incidence	0.790 (0.624–1.001)	[74]

**Table 3:** Outcomes and prognosis in patients with gastric cancer and diabetes mellitus.

Outcomes and Prognosis	Explanations
1. Higher incidence of second primary malignancy	A 15-year follow-up study in Taiwan suggests that diabetes patients with GCa have a significantly higher incidence of second primary malignancy than non-diabetes patients with GCa.
2. Deterioration of hyperglycemia and infection at GCa diagnosis	Patients with diabetes mellitus may develop poorer glycemic control, requiring the use of insulin, and have an increased risk of tuberculosis infection when they are diagnosed with GCa.
3. Poorer prognosis	GCa patients with pre-existing diabetes or who develop postoperative hyperglycemia may have more postoperative complications and poorer survival.
4. Remission of diabetes mellitus or improved glycemic control	Patients with diabtetes and GCa who receive gastrectomy may have remission of diabetes mellitus or improved glycemic control, especially in patients receiving Billroth II reconstruction or Roux-en-Y gastric bypass. The remission of diabetes mellitus and improvement of metabolic control may be because of increased insulin secretion and improved insulin resistance resulting from reduced appetite and loss of body weight and changes in gut hormones and gut microbiota after Billroth II or Roux-en-Y gastric bypass.
5. More complications after gastrectomy or chemotherapy	Diabetes patients with GCa suffer from more complications after gastrectomy or chemotherapy. Increased complications may include gallstones, anemia, intra-abdominal infection, osteoporosis/fracture, pulmonary infection, anastomotic leakage, thrombocytopenia, acute kidney injury, etc. Diabetes patients with GCa may require prolonged stays in intensive care units, more readmission and unplanned reoperation.

GCa: gastric cancer.

**Table 4:** Potential mechanisms of metformin against gastric cancer mentioned in the literature.

Potential Mechanisms	References
Inhibition of a signaling pathway involving hypoxia-inducible factor 1α/M2 isoform of pyruvate kinase	[211]
2. Inhibition of the Shh signaling pathway through AMPK	[212]
3. Suppression of metastatic traits of EMT	[213]
4. Stimulating calmodulin-like protein 3 secretion from gastric tumor-associated fibroblasts	[214]
5. Promoting beclin 1-dependent autophagy	[215]
6. Triggering the intrinsic apoptotic response via activating AMPK and suppressing mTOR/protein kinase B signaling	[216]
7. Targeting GCa stem cells	[217]
8. Inhibiting angiogenesis and metastasis involving the protein tyrosine phosphatase receptor delta-C-X-C motif chemokine ligand 8 axis	[218]
9. Inhibiting invasion via downregulating long non-coding RNA H19	[219]
10. Increasing acid-secreting parietal cells from stem cells via AMPK-dependent pathways	[220]
11. Inhibiting the transcription factor HNF4α via downregulation of *KLF5/GATA4/GATA6* oncogenes	[221]
12. Inducing apoptosis via inhibition of survivin mediated by AMPK/mTOR	[222]
13. Blocking cell cycle in G_0_–G_1_ by decreasing G_1_ cyclins and reducing the phosphorylation of epidermal growth factor receptor and insulin-like growth factor 1 receptor, probably via altering miRNAs expression	[223]
14. Modulating metastatic capacity by reducing the expression of vascular endothelial growth factor and blocking EMT	[224]
15. Decreasing the expression of EMT and stemness markers and reducing spheroid formation	[225]
16. Increasing the expression of phosphorylated form of acetyl-CoA carboxylase	[226]
17. Downregulating specificity protein transcription factors	[227]
18. Via the AMPKα-HNF4α-WNT5A signaling cascade	[228]
19. Suppressing HP-induced apoptosis	[229]
20. Downregulating B-lymphoma Moloney murine leukemia virus insertion region-1, an oncogene, via AMPK	[230]
21. Activating calcium binding protein 39-like, a tumor suppressor gene, via AMPK	[231]
22. Inhibiting in vivo tumor xenograft metastasis to peritoneum, probably via inhibition of nuclear factor-ĸB expression	[232]
23. Inhibiting N-methyl-N-nitrosourea induced GCa development in db/db mice	[233]
24. Interaction with chemotherapeutic drugs	[227,234,235,236,237]
25. Reduction of HP infection	[238]
26. Improving insulin resistance and reducing inflammation	[239]

AMPK: 5′ adenosine monophosphate-activated protein kinase; EMT: epithelial-mesenchymal transition; GCa: gastric cancer; HNF4α: hepatocyte nuclear factor 4 alpha; HP: *Helicobacter pylori*; mTOR: mammalian target of rapamycin.
